# Self-compassion and psychological help-seeking delay: an indirect association through mental health problems and negative academic emotions

**DOI:** 10.3389/fpsyt.2026.1889435

**Published:** 2026-08-19

**Authors:** Xiaohang Wang, Xingfa Long, Xinyuan Zhang, XinJing Wang

**Affiliations:** 1 Department of Human Development and Family Studies, Faculty of Human Ecology, Universiti Putra Malaysia, Serdang, Malaysia; 2 School of Public Administration, Hohai University, Nanjing, China; 3 Quzhou College of Technology, Quzhou, China; 4 School of Psychology, Nanjing Normal University, Nanjing, China; 5 United Front Work Teaching and Research Section, Party School of the Rizhao Municipal Committee of the Communist Party of China, Rizhao, China

**Keywords:** academic emotions, help-seeking delay, psychological distress, self-compassion, university students

## Abstract

**Objective:**

This study aimed to examine the associations among self-compassion, mental health status, negative academic emotions, and psychological help-seeking delay among college students, with particular attention to the indirect associations involving mental health status and negative academic emotions.

**Methods:**

A convenience sampling method was used to survey 753 college students. Data were collected using the Self-Compassion Scale, General Health Questionnaire, Negative Academic Emotions Scale, and Psychological Help-Seeking Delay Scale. Structural equation modeling was conducted to examine the proposed pattern of associations among the study variables, and indirect associations were evaluated using bootstrapping procedures.

**Results:**

Significant correlations were found among the key variables (*p*s < 0.001). Analyses of indirect associations revealed significant indirect associations involving mental health problems and negative academic emotions in the association between self-compassion and psychological help-seeking delay. The total effect between self-compassion and psychological help-seeking delay was significant (*β* = -0.46, 95% CI [-0.527, -0.391]). Specifically, significant indirect associations were observed involving mental health problems (*β* = -0.09, 95% CI [-0.185, -0.022]) and negative academic emotions (*β* = -0.13, 95% CI [-0.240, -0.056]). Furthermore, a significant sequential indirect association involving mental health problems and negative academic emotions was identified (*β* = -0.10, 95% CI [-0.189, -0.054]).

**Conclusion:**

Self-compassion was directly associated with psychological help-seeking delay among college students and was also indirectly associated with help-seeking delay through mental health problems and negative academic emotions, both independently and sequentially. Given the cross-sectional design, these findings should be interpreted as associational rather than causal, and longitudinal studies are needed to confirm the proposed directional relationships among these variables.

## 1 Introduction

College students represent a key population in the emerging adulthood stage and constitute an important human resource for future social development. As they transition from adolescence to adulthood, individuals are required to navigate multiple developmental tasks, including identity formation, autonomous development, and academic as well as social adaptation challenges (1, 2). Alongside rapid societal changes, college students are facing increasing pressures and challenges, and their mental health status has become a growing concern (3). A meta-analysis conducted by Chen et al. (4) revealed that the prevalence rates of depression and anxiety among Chinese college students were 20.80% and 13.70%, respectively, with an overall increasing trend over time.

To address these issues, universities have invested in and promoted subsidized or free counseling and psychological support services for students. However, despite the widespread availability of such services, there remains a striking discrepancy between high prevalence rates of psychological distress and low utilization of professional help-seeking services among college students (5). Timely access to professional psychological intervention has been shown to effectively alleviate mental health problems among individuals experiencing psychological distress or disorders (6). Professional psychological help-seeking refers to the behavior of seeking assistance from trained professionals or institutions to address mental illness, personal issues, emotional problems, or to reduce psychological distress (7, 8). Nevertheless, only a small proportion of college students actively seek professional psychological help when needed (5, 9), and many tend to rely instead on self-regulation or informal support from friends to cope with emotional difficulties (10, 11).

More importantly, a considerable number of students delay or postpone seeking professional psychological help even when experiencing significant distress. This phenomenon is referred to as psychological help-seeking delay (12). Individuals exhibiting help-seeking delay may initially consider professional psychological services but ultimately postpone or abandon seeking help. Empirical evidence suggests that, on average, individuals often experience more than one year between the onset of psychological problems and actual help-seeking (13, 14). During this period, individuals may attempt to resolve problems independently or seek alternative forms of support, with professional psychological counseling often being considered as a last resort (15). Recent evidence further indicates that emotional avoidance and poorer mental health status are associated with a lower likelihood of initiating professional help-seeking (16). Prolonged help-seeking delay is associated with greater psychological distress and poorer psychological outcomes (17). Therefore, identifying factors that reduce psychological help-seeking delay and promote timely help-seeking is of critical importance.

Self-compassion, introduced within the framework of positive psychology, refers to an adaptive self-attitude characterized by treating oneself with kindness and understanding in the face of failure, inadequacy, or suffering, rather than engaging in self-criticism (18, 19). According to Neff, self-compassion comprises three components: self-kindness, common humanity, and mindfulness, which are thought to promote self-acceptance and psychological well-being. A growing body of research has demonstrated that self-compassion is closely associated with mental health outcomes (20–22). Individuals with higher levels of self-compassion are more likely to engage in self-care and may place greater importance on their psychological well-being, which has been associated with a greater willingness to seek external help for emotional difficulties (23). Those who treat themselves with kindness and perceive suffering as part of shared human experience are more likely to seek professional psychological assistance, a finding supported by studies among college student and male populations (24, 25). In contrast, individuals with low self-compassion tend to engage in excessive self-criticism and emotional concealment, which may prolong the delay between problem recognition and professional help-seeking (26). Therefore, self-compassion may be an important factor associated with psychological help-seeking delay.

### 1.1 The role of mental health problems

Previous studies have identified mental health problems as a key determinant of help-seeking behavior (6, 27). Adolescents and young adults experiencing higher levels of psychological distress are less likely to utilize formal mental health services or express intentions to seek professional help (28, 29), whereas individuals with better mental health status are more likely to engage in help-seeking behaviors (30). According to the Theory of Planned Behavior (TPB, 31), help-seeking intention is determined by attitudes toward the behavior, subjective norms, and perceived behavioral control. Psychological distress may weaken perceived behavioral control and reinforce negative attitudes toward help-seeking, thereby reducing help-seeking intention (32).

Higher levels of self-compassion are typically associated with better mental health, reflected in lower levels of anxiety and depressive symptoms. This may be because self-compassion enables individuals to respond to stress and negative emotions with understanding and acceptance rather than self-judgment (33, 34). Better mental health has been associated with more adaptive coping strategies, lower avoidance tendencies, and a greater likelihood of seeking professional psychological help when facing emotional or psychological difficulties.

### 1.2 The role of negative academic emotions

Learning is an important part of university life and plays a crucial role in enhancing students’ professional skills and personal development. Academic emotions, as non-intellectual factors, are of significant importance for students’ learning outcomes (35).

Reinhard Pekrun proposed the Control-Value Theory of Achievement Emotions, which first explicitly defined the concept of academic emotions (36). The Control-Value Theory defines academic emotions as various emotions directly linked to learning activities and outcomes, including enjoyment, hope, pride, relief, anger, anxiety, shame, hopelessness, and boredom (37), thus encompassing emotions experienced during the learning process within the scope of academic emotions. Negative academic emotions have been associated with problems such as lack of concentration (35) and learning fatigue (38) among university students. Studies have found that individuals with high levels of self-compassion have a more accurate understanding of their shortcomings and faults. They tend to view themselves with care and compassion rather than harsh self-criticism. Accordingly, individuals with higher levels of self-compassion tend to report less fear of failure and stronger perceptual abilities (39). Therefore, individuals with higher levels of self-compassion are more likely to adopt communication and adaptive help-seeking strategies during learning, which has been associated with lower levels of hesitation when seeking help (40).

### 1.3 The role of mental health problems and negative academic emotions

Furthermore, it is crucial to consider whether mental health status is related to students’ academic emotions. A growing body of research has consistently demonstrated that mental health is closely related to students’ academic performance and emotional experiences in educational contexts. University students with higher levels of psychological well-being tend to report more positive academic emotions, greater learning engagement, and fewer negative emotional experiences during learning activities. For example, students with higher psychological resilience and well-being are more likely to experience enjoyment, hope, and academic confidence, while exhibiting lower levels of anxiety and emotional distress (41, 42). Therefore, self-compassion may represent an important psychological resource that is associated with better mental health, more positive psychological resources, more adaptive responses to academic setbacks, and a greater willingness to seek support when encountering difficulties. In addition, higher levels of self-compassion have been associated with better mental health outcomes (43), fewer depressive symptoms (44), and lower levels of hopelessness and suicide risk (45).

### 1.4 The present study

This paper aims to explore the underlying connections between college students’ self-compassion, mental health problems, negative academic emotions, and psychological help-seeking delay, providing theoretical and practical bases for improving college students’ mental health and learning conditions, and enhancing psychological help-seeking behaviors.

Building on the theoretical background and literature reviewed, we formulated four research hypotheses:

H1: Self-compassion is expected to be negatively associated with psychological help-seeking delay.

H2: Mental health problems are expected to show an indirect association between self-compassion and psychological help-seeking delay.

H3: Negative academic emotions are expected to show an indirect association between self-compassion and psychological help-seeking delay.

H4: A serial indirect association involving mental health problems and negative academic emotions is expected between self-compassion and psychological help-seeking delay.

**Figure 1:**
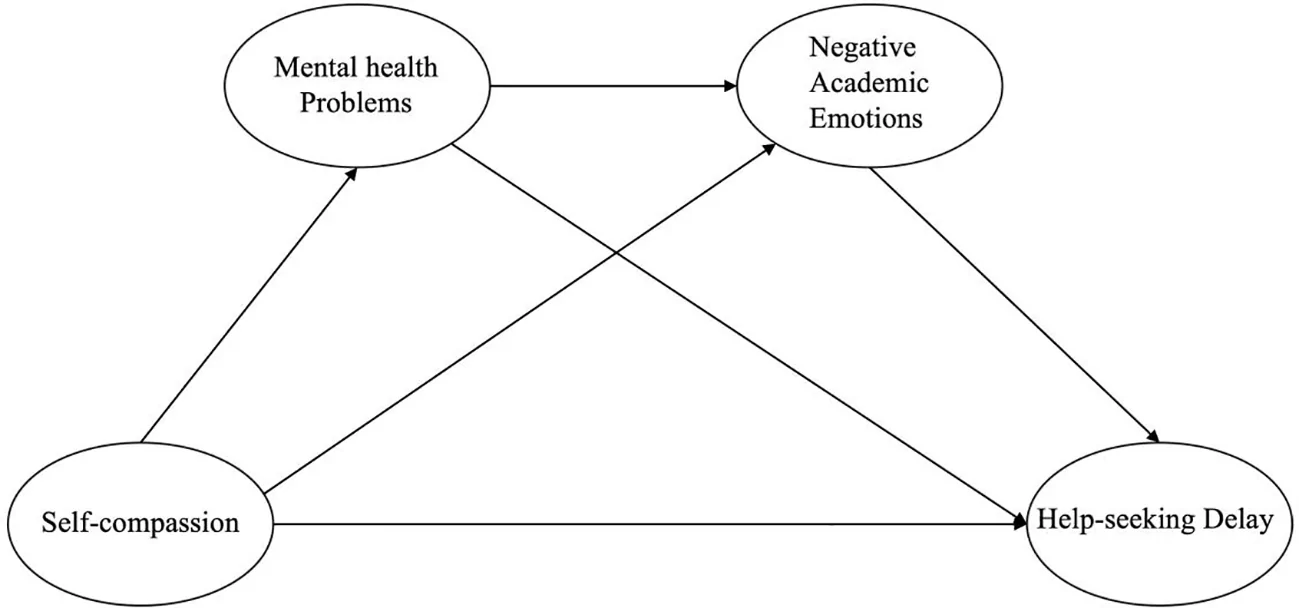
Theoretical model diagram.

These hypotheses are graphically represented in **Figure 1**, providing a clear visual framework of the expected relationships and interactions among the variables studied.

## 2 Methods

### 2.1 Participants and procedure

A total of 827 college students from a university in Jiangsu Province, China, were surveyed through convenience sampling. The final sample consisted of 753 valid responses, yielding a questionnaire validity rate of 91.01%. This study employed the Chinese online survey platform Wen Juan Xing (https://www.wjx.cn/) to disseminate questionnaires. The research objectives were elucidated at the onset of the survey, ensuring confidentiality, anonymity, and emphasizing the sole utilization of responses for research endeavors. All participants voluntarily engaged in completing the questionnaire, with explicit consent and the liberty to withdraw from the study at will. The informed consent document was provided in conjunction with the survey questionnaire. Following a comprehensive review of the informed consent document and a thorough understanding of the study’s objectives and potential risks, participants voluntarily contributed to the survey anonymously. The survey adhered to the principles outlined in the revised Helsinki Declaration of 1989 and received approval from the institutional ethics committee.

Among the surveyed participants, there were 482 males (64.01%) and 271 females (35.99%). Regarding academic years, there were 270 freshmen (35.86%), 94 sophomores (12.48%), 160 juniors (21.25%), 95 seniors (12.62%), and 134 graduate students or above (17.80%). Among all surveyed participants, 630 individuals (83.67%) believed they did not need psychological assistance, while 123 individuals (16.33%) believed they needed psychological assistance. Among those who believed they needed psychological assistance, 45 individuals had never considered using psychological assistance, 52 individuals had considered seeking help but had not taken action, and 26 individuals had participated in or were currently undergoing psychological assistance.

### 2.2 Material and methods

#### 2.2.1 Self-compassion

The Chinese version of the Self-Compassion Scale aims to assess self-compassion levels among current university students (46). The scale consists of 12 items, divided into three dimensions: self-kindness, common humanity, and mindfulness. It employs a 5-point Likert scale for scoring, where college students rate items (e.g., “When I encounter difficulties, I tend to see them as part of the human experience.”) from 1 (Never) to 5 (Always). A higher score indicates a higher level of self-compassion. In this study, the Cronbach’s alpha coefficient of the scale was 0.93, and the alpha coefficients for each dimension ranged from 0.81 to 0.90, indicating high reliability.

#### 2.2.2 Psychological help-seeking delay

The Help-Seeking Delay Scale was used to measure the extent of psychological help-seeking delay among college students (12). The scale comprises 6 items, defining help-seeking delay as the degree of hesitation and reluctance individuals experience when considering seeking help from mental health professionals or institutions. College students rate these items (e.g., “There are always reasons why I hesitate to seek help from mental health professionals”) on a 5-point Likert scale from 1 (Strongly Disagree) to 5 (Strongly Agree). A higher score indicates a higher degree of hesitation in deciding to seek professional psychological help and a longer time required to make the decision for help-seeking. In this study, the Cronbach’s alpha coefficient of the scale was 0.95.

#### 2.2.3 Mental health problems

This study utilized the depression and anxiety subscales from the General Health Questionnaire (GHQ) to measure mental health status (47). The depression subscale comprises 6 items, while the anxiety subscale comprises 5 items. Example items include “*Losing confidence in oneself*” (depression) and “*Feeling restless and nervous all day long*” (anxiety). Following the original scoring procedure of the GHQ and prior studies involving Chinese university students (47–49), the present study adopted the dichotomous scoring method, with responses of “Yes” coded as 1 and “No” coded as 0. This scoring approach represents the standard procedure for using the GHQ as a screening instrument. Previous research has demonstrated that, in certain research contexts, dichotomous scoring yields more stable psychometric properties and lower measurement error than the Likert scoring method (50). Furthermore, this dichotomous scoring approach has been widely adopted in recent empirical studies employing the GHQ (51, 52). A higher score indicates higher levels of depression and anxiety, reflecting more mental health problems. The Cronbach’s alpha coefficient of the scale in this study was 0.83.

#### 2.2.4 Negative academic emotions

This study employed the academic anxiety and academic boredom subscales from the Academic Emotion Questionnaire to measure negative academic emotions (53). The academic anxiety subscale comprises 7 items (e.g., “I feel nervous and anxious before exams”), while the academic boredom subscale comprises 11 items (e.g., “I feel studying is pointless”). Items are rated on a 5-point Likert scale ranging from 1 (“Never”) to 5 (“Always”), with higher scores indicating higher levels of academic anxiety and boredom. The Cronbach’s alpha coefficients for the academic anxiety and academic boredom subscales were 0.92 and 0.94, respectively.

### 2.3 Statistical analysis

SPSS version 23.0 was used for statistical analysis. Descriptive statistics were employed to summarize general demographic data and conducting a normality test during data processing. Count data were presented as *n* (%), while measurement data were expressed as mean ± standard deviation (SD). Pearson correlation analysis was conducted to explore the correlations between variables, and analysis of variance (ANOVA) was used to compare the scores of self-compassion, mental health problems, negative academic emotions, and help-seeking delay among students with different demographic characteristics. The associations among the study variables were examined using structural equation modeling (SEM) with AMOS 23.0, with a significance level set at *p* < 0.05.

## 3 Results

### 3.1 Common method biases

Anonymous data collection, reverse scoring, and other methods were employed to reduce common method bias during the questionnaire collection process. In the data analysis stage, the Harman single-factor method was used to test for common method bias. Exploratory factor analysis was conducted on all questionnaire items without rotation, revealing six factors with eigenvalues greater than 1. The explained variance of the first factor was 31.68%, which is below the critical threshold of 40%, suggesting that common method bias was unlikely to substantially affect the findings (54).

### 3.2 Descriptive statistics and correlations

Pearson correlation analyses were conducted to examine the associations among self-compassion, mental health status, academic anxiety and boredom, and psychological help-seeking delay. The results indicated that self-compassion was significantly negatively correlated with other variables (*p* < 0.01), while anxiety, depression, academic anxiety and boredom, and help-seeking delay were all significantly positively correlated with each other (*p* < 0.01). The means, *SD*s and Pearson correlations for the study variables were reported in **Table 1**.

**Table 1:** Descriptive statistics and correlations among variables of interest.

Variables	*M*	*SD*	Skewness	Kurtosis	1	2	3	4	5	6
1. Self-compassion	11.32	2.17	-0.86	-0.28	-					
2. Anxiety	0.90	1.43	1.57	1.37	-0.34^**^	-				
3. Depression	0.74	1.24	1.37	5.31	-0.35^**^	0.57^**^	-			
4. Academic anxiety	22.32	6.67	-0.41	-0.13	-0.36^**^	0.34^**^	0.30^**^	-		
5. Academic boredom	23.92	9.06	0.64	0.33	-0.39^**^	0.34^**^	0.41^**^	0.44^**^	-	
6. Help-seeking Delay	12.63	6.09	0.61	-0.46	-0.43^**^	0.41^**^	0.37^**^	0.39^**^	0.41^**^	-

^**^ *p* < 0.01, the same below.

### 3.3 Demographic difference analyses

To examine the potential influence of the unbalanced gender distribution, independent-samples t tests were conducted to compare male and female participants on the major study variables. The results indicated no significant gender differences in self-compassion, depression, anxiety, academic anxiety, academic Boredom, or psychological help-seeking delay (all *p*s >.05). Moreover, the effect sizes were negligible (Cohen’s *d*s ranged from 0.04 to 0.09), suggesting minimal gender-related differences in the study variables.

**Table 2** shows significant differences in scores on various questionnaires among students of different grades (*p* < 0.05). The self-compassion level of fourth-year students is significantly lower than that of other grades (*p* < 0.05); the anxiety level of first-year students differs significantly from that of third and fourth-year students; and the depression level of first-year students differs significantly from that of fourth-year students. *Post-hoc* tests showed that freshmen reported significantly higher self-compassion than juniors and seniors (*p*s <.05). Graduate students also reported significantly higher self-compassion than juniors and seniors (*p*s <.05). No other significant differences were found; for psychological help-seeking delay scores, *post-hoc* tests indicated that seniors reported significantly higher psychological help-seeking delay than freshmen, sophomores, and graduate students (*p*s <.05). Juniors also reported higher help-seeking delay than sophomores and graduate students (*p*s <.05). The detailed data are presented in **Table 2**.

**Table 2:** Mean scores, standard deviations, and ANOVA results for psychological variables across grade levels.

Grade level	*n*	Self-compassion	Anxiety	Depression	Academic anxiety	Academic boredom	Help-seeking Delay
Freshman	270	11.72 ± 2.05	0.64 ± 1.21	0.69 ± 1.19	23.43 ± 6.76	22.01 ± 8.22	12.84 ± 6.42
Sophomore	94	11.39 ± 2.13	0.95 ± 1.62	0.67 ± 1.20	21.71 ± 7.25	25.57 ± 9.64	11.25 ± 5.50
Junior	160	11.01 ± 2.27	1.08 ± 1.44	0.79 ± 1.26	23.11 ± 5.61	26.19 ± 8.96	13.28 ± 5.91
Senior	95	10.27 ± 2.26	1.45 ± 1.80	1.19 ± 1.57	23.50 ± 6.45	27.97 ± 10.23	13.53 ± 5.94
Graduate student	134	11.56 ± 2.01	0.81 ± 1.24	0.53 ± 0.99	18.75 ± 6.16	21.03 ± 7.56	11.78 ± 5.94
*F*		9.38	6.76	4.44	13.84	15.57	2.94
*p*		< 0.01	< 0.01	< 0.01	< 0.01	< 0.01	< 0.05

### 3.4 Structural equation modeling and indirect effect analysis

This study used AMOS software to conduct structural equation modeling analysis, exploring the serial indirect associations between self-compassion and psychological help-seeking delay. The results of the indirect association model analysis indicated a good model fit: *χ*^2^/*df* = 2.87 (*p* < 0.01), GFI = 0.99, NFI = 0.98, CFI = 0.99, TLI = 0.98, RMSEA = 0.05(90% CI=[0.03,0.07]), indicating a good model fit. The significance of the indirect associations was examined using the bootstrap method, with 5,000 bootstrap resamples drawn from the original data to estimate the 95% confidence intervals for the indirect associations.

**Figure 2:**
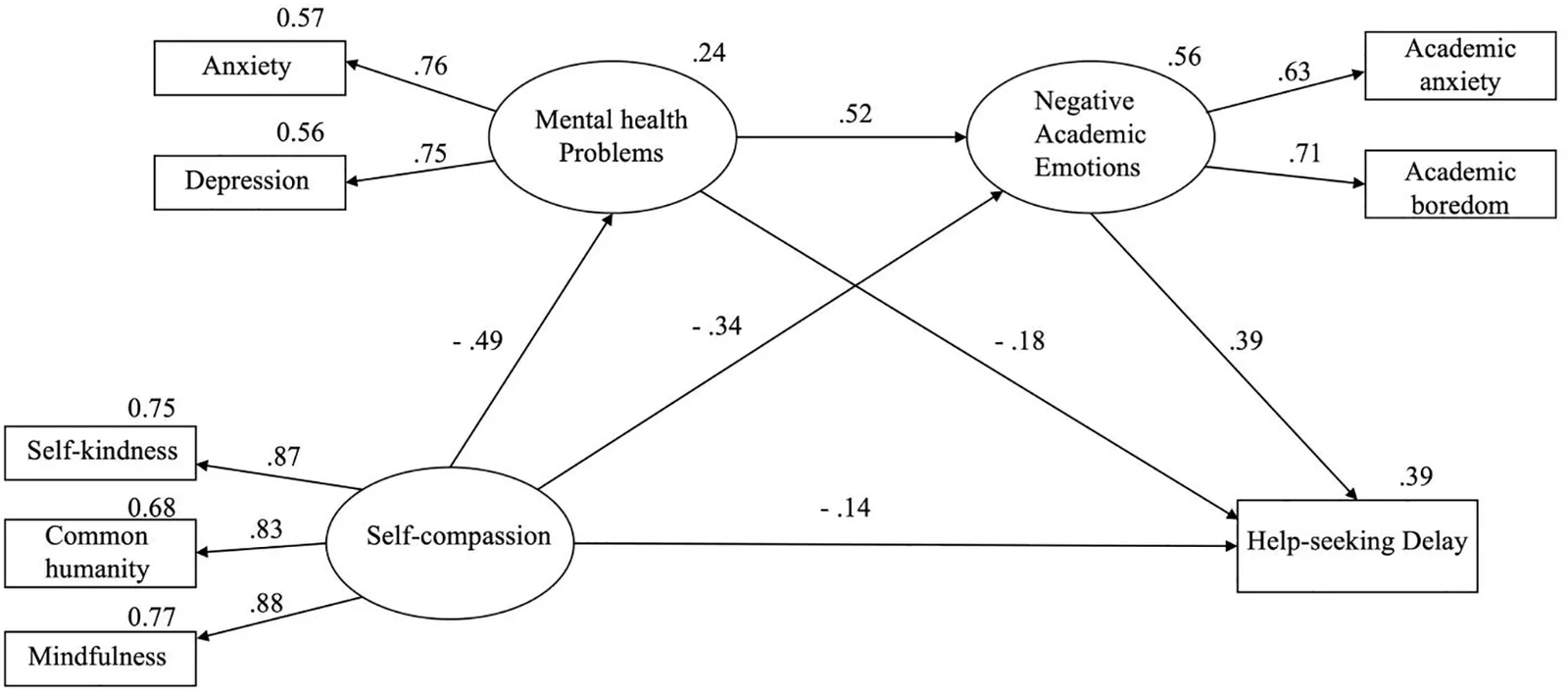
Structural model with standardized estimates.

The results indicate that, as shown in **Figure 2**: (1) Self-compassion was negatively associated with mental health problems, while mental health problems were positively associated with psychological help-seeking delay. An indirect association through mental health problems was observed between self-compassion and help-seeking delay. (2) Self-compassion is negatively associated with negative academic emotions, while negative academic emotions are positively associated with psychological help-seeking delay. (3) Self-compassion was negatively associated with mental health problems, while mental health problems were positively associated with negative academic emotions. Negative academic emotions were, in turn, positively associated with psychological help-seeking delay. A significant direct association was also observed between self-compassion and psychological help-seeking delay. In summary, mental health problems and negative academic emotions were found to account for part of the association between self-compassion and help-seeking delay. Indirect effects represented 70.07% of the total statistical effect. Proportions were calculated based on standardized estimates relative to the total effect. The indirect effects are presented in **Table 3**.

**Table 3:** Direct, indirect, and total effects in the indirect association model.

Effect type	Pathway	Estimate	*SE*	*Z*	95% CI (Bias-corrected)	95% CI (Percentile)
Total effect	Self-compassion → Help-seeking delay	-0.46	0.04	-12.81	[-0.527, -0.391]	[-0.528, -0.392]
Direct effect	Self-compassion → Help-seeking delay	-0.14	0.06	-2.49	[-0.240, -0.022]	[-0.240, -0.023]
Indirect effect 1	Self-compassion → Mental health problems → Help-seeking delay	-0.09	0.04	-2.28	[-0.185, -0.022]	[-0.180, -0.018]
Indirect effect 2	Self-compassion → Negative academic emotions → Help-seeking delay	-0.13	0.05	-2.89	[-0.240, -0.056]	[-0.231, -0.050]
Indirect effect 3	Self-compassion → Mental health problems → Negative academic emotions → Help-seeking delay	-0.10	0.03	-3.13	[-0.189, -0.054]	[-0.174, -0.049]
Total indirect effect	Combined indirect pathways	-0.32	0.04	-7.51	[-0.423, -0.251]	[-0.418, -0.248]

*SE*, standard error; *CI*, confidence interval. Bootstrapped confidence intervals were estimated using 5,000 resamples. Indirect effects represent specific indirect association pathways within the model. Proportions of effects are not shown in this table but were calculated separately as ratios of indirect and direct effects to the total effect. All confidence intervals are based on bias-corrected and percentile bootstrap methods.

## 4 Discussion

### 4.1 Differences of variables in demographics

This study found significant differences in self-compassion, mental health problems, negative academic emotions, and psychological help-seeking delay among different academic years. Senior undergraduate students are in a critical developmental period, which may be associated with the emergence of anxiety and depression due to practical issues such as postgraduate study, employment, and future planning (55–57). Consequently, seniors exhibit lower levels of self-compassion and psychological well-being. Meanwhile, third- and fourth-year students often engage in various extracurricular activities, research projects, and internships, which may dilute their focus on academic learning and contribute to increased negative academic emotions (58, 59). Hence, senior undergraduate students exhibit lower levels of self-compassion and psychological well-being. Additionally, senior students may perceive themselves as more adaptable to university life and more capable of handling their own issues, while fearing that seeking help may undermine their image as senior students (60), thereby increasing the likelihood of procrastination in seeking psychological help.

### 4.2 The relationship between self-compassion and psychological help-seeking delay

The present study found that self-compassion was negatively associated with psychological help-seeking delay among university students, supporting Hypothesis 1. Specifically, students with higher levels of self-compassion exhibited lower psychological help-seeking delay when experiencing psychological distress. This finding is consistent with previous research demonstrating that greater self-compassion is associated with more adaptive coping strategies and a greater willingness to seek external support when facing difficulties (25, 61).

Self-compassion comprises three core components: self-kindness, common humanity, and mindful awareness of negative emotions rather than avoidance (19). These characteristics help reduce self-criticism and shame (61), both of which have been consistently identified as major psychological barriers to professional psychological help-seeking (62). Consequently, individuals with higher self-compassion may be better able to acknowledge their psychological difficulties without excessive self-blame and are more likely to perceive seeking psychological support as an adaptive coping strategy rather than as an indication of personal inadequacy.

The present findings can also be interpreted within the framework of the TPB. Although TPB proposes that behavior is most directly associated with by behavioral intention, Ajzen (31, 63) argued that background factors may indirectly influence behavior through their effects on attitudes, subjective norms, and perceived behavioral control. From this perspective, self-compassion may function as an important psychological background factor that associated with timely psychological help-seeking. Specifically, individuals with higher levels of self-compassion may develop more favorable attitudes toward professional psychological services because they are less likely to interpret help-seeking as a sign of personal failure or incompetence (25, 64). In addition, self-compassion may enhance perceived behavioral control by increasing individuals’ confidence in acknowledging their emotional difficulties and in seeking appropriate professional support despite concerns about stigma or negative evaluation. These more adaptive cognitive appraisals may subsequently strengthen help-seeking intentions (63, 65), thereby reducing the likelihood of delaying professional psychological help-seeking.

Notably, the present findings suggest that self-compassion may serve as an antecedent psychological protective resource associated with psychological help-seeking delay. However, given the cross-sectional nature of the present study, these interpretations should be considered preliminary and should not be construed as evidence of causal relationships. Future longitudinal and experimental studies are needed to clarify the temporal ordering and causal associations among these variables.

### 4.3 Mental health problems in the relationship between self-compassion and psychological help-seeking delay

The present study identified a significant indirect association of self-compassion with psychological help-seeking delay through mental health problems. Specifically, higher levels of self-compassion were associated with fewer symptoms of anxiety and depression, which were in turn associated with lower levels of psychological help-seeking delay. This supports Hypothesis 2. These findings further suggest that better general mental health may help explain the association between self-compassion and more timely professional psychological help-seeking (33, 66).

Self-compassion, as a protective psychological resource, may be associated with the way individuals regulate psychological distress by encouraging them to respond to personal setbacks with kindness, acceptance, and balanced awareness rather than excessive self-criticism and emotional avoidance (19). Consequently, higher levels of self-compassion have been associated with lower levels of anxiety and depression (67, 68). Lower levels of psychological distress may also be associated with greater availability of cognitive and emotional resources, enabling individuals to recognize their emotional difficulties more objectively and adopt more adaptive coping responses, which may in turn be associated with lower levels of psychological help-seeking delay (68, 69).

This interpretation is also consistent with the TPB. Although mental health problems are not a core component of the TPB, Ajzen (31, 63) proposed that background factors may shape individuals’ beliefs and thereby be associated with behavioral outcomes (70). Better mental health may be associated with fewer maladaptive beliefs, such as catastrophizing, hopelessness, and self-stigma, which have been linked to reluctance to seek professional psychological support (71, 72). Conversely, students experiencing more severe anxiety and depression may be more likely to perceive psychological services as less accessible, less effective, or more threatening, and these perceptions may be associated with less favorable attitudes toward psychological help-seeking and lower perceived behavioral control (73). Such cognitive evaluations may, in turn, be associated with greater psychological help-seeking delay (74).

Although mental health problems were associated with the relationship between self-compassion and psychological help-seeking delay, this association was only partial, suggesting that other psychological processes may also be involved. These findings suggest that better general mental health may represent one important aspect of more timely psychological help-seeking, whereas subsequent emotional and cognitive processes may also be associated with whether improvements in mental health are reflected in actual help-seeking decisions. This interpretation provided the rationale for the subsequent examination of negative academic emotions as a second-stage variable in the relationship between self-compassion and psychological help-seeking delay.

### 4.4 Negative academic emotions in the relationship between self-compassion and psychological help-seeking delay

This study found a significant indirect association involving negative academic emotions between self-compassion and psychological help-seeking delay, supporting Hypothesis 3. Specifically, students with higher levels of self-compassion reported fewer negative academic emotions, which, in turn, were associated with lower levels of psychological help-seeking delay. This finding extends previous research by suggesting that self-compassion may be associated with psychological help-seeking not only through its relationship with general mental health but also through its association with students’ emotional experiences in academic contexts (66, 75).

According to the control-value theory, students’ academic emotions are primarily are closely related to their perceived level of control over academic activities and the value they attach to academic outcomes (37). When students perceive limited control or experience excessive academic demands, they are more likely to report negative achievement emotions, such as anxiety, frustration, and boredom (76). Within this framework, self-compassion may function as a psychological resource that encourages more adaptive self-evaluation and reduces excessive self-criticism when students encounter academic setbacks (39, 77). Consequently, students with higher levels of self-compassion may be more likely to perceive academic challenges as manageable rather than threatening, thereby reporting fewer negative academic emotions.

In addition, heightened negative academic emotions may be associated with greater emotional avoidance and reduced motivation to seek psychological support, thereby contributing to psychological help-seeking delay. Students experiencing persistent negative academic emotions may prefer to cope with their distress on their own, fear being judged because of their psychological difficulties, or perceive help-seeking as a sign of personal inadequacy (78, 79). As a result, they may postpone seeking professional psychological services even when experiencing substantial psychological distress (80). In contrast, students experiencing fewer negative academic emotions may be more aware of their psychological needs and more likely to seek professional support in a timely manner.

Taken together, the present findings suggest that negative academic emotions represent an important emotional mechanism linking self-compassion and psychological help-seeking delay, thereby extending the existing literature. Previous studies have primarily focused on the direct protective role of self-compassion in emotional well-being, whereas the present findings highlight the potential importance of academic emotional experiences in students’ decisions regarding when to seek professional psychological support.

### 4.5 Sequential indirect associations through mental health problems and negative academic emotions

The present study further identified a significant serial indirect association through mental health problems and negative academic emotions in the association between self-compassion and psychological help-seeking delay, supporting Hypothesis 4. Specifically, higher levels of self-compassion were associated with lower levels of anxiety and depression, which, in turn, were related to reduced negative academic emotions and ultimately to lower psychological help-seeking delay. Compared with examining a single indirect association, the serial indirect association model allows for the examination of sequential associations among self-compassion, mental health problems, negative academic emotions, and psychological help-seeking delay.

Individuals with higher levels of self-compassion are more likely to respond to psychological distress with acceptance and emotional balance rather than self-criticism and avoidance, thereby alleviating symptoms of anxiety and depression (18, 19, 33, 67). As overall mental health improves, students may become better equipped to cope with academic demands and are therefore less likely to experience persistent academic anxiety or academic boredom (37). As negative academic emotions diminish, students may become less likely to avoid confronting their psychological difficulties and more willing to recognize the need for professional psychological support before their problems become more severe. These findings suggest that psychological help-seeking delay is not attributable to a single psychological factor but rather reflects a progressive psychological process, in which improvements in general psychological functioning are associated with more adaptive emotional experiences in academic contexts, and these experiences are in turn associated with help-seeking decisions.

Furthermore, the present findings extend the application of the Theory of Planned Behavior by suggesting that improvements in mental health and academic emotional experiences may contribute to more adaptive evaluations of professional psychological help-seeking, thereby reducing the tendency to delay seeking help (31, 63). Psychological distress, excessive self-reliance, and stigma have been identified as important barriers to timely help-seeking among university students, whereas greater awareness of psychological needs and more adaptive attitudes toward professional psychological services facilitate help-seeking (81).

Overall, the present findings extend the existing literature by further revealing the hierarchical psychological process underlying psychological help-seeking delay. This hierarchical model suggests that the influence of self-compassion on psychological help-seeking may operate through successive stages of psychological adjustment, beginning with general psychological functioning, progressing to emotional experiences in specific academic contexts, and ultimately influencing behavioral decision-making (37, 82).

### 4.6 Limitations and future directions

This study found that self-compassion is negatively associated with psychological help-seeking delay, and explored the correlational pathways among college students experiencing mental health difficulties and negative academic emotions. However, it is worth noting that this study still has limitations.

Firstly, the study employed a cross-sectional design, which only allows for the identification of correlations among self-compassion, mental health problems, negative academic emotions, and psychological help-seeking delay, without establishing causality. Future research could utilize various methodologies, such as experimental or longitudinal designs, to explore the dynamic nature of the relationship between self-compassion and psychological help-seeking delay among college students. Additionally, the present study assessed anxiety and depression using the two-point scoring version of the General Health Questionnaire, which may have underestimated the true associations among the study variables. Future research could employ a continuous scoring approach to assess these constructs, thereby providing a more accurate examination of the relationships among the variables.

Secondly, this study primarily relied on self-report measures, which may be subject to social desirability effects and common method bias. Participants may provide responses that align with social expectations, potentially affecting the objectivity of the findings. Moreover, the use of a single data source may limit the interpretability of the relationships among variables. Future studies are encouraged to incorporate multiple data sources, behavioral indicators, or longitudinal designs to improve the robustness of the findings.

Thirdly, although no significant gender differences were observed in the study variables, the sample included a higher proportion of male participants than female participants. Therefore, caution is warranted when generalizing the findings to populations with different gender compositions. Future studies should recruit more gender-balanced samples and further examine whether the observed associations are consistent across gender.

Finally, this study did not examine other potential factors that may be associated with the relationship between self-compassion and psychological help-seeking delay. Previous research has suggested that factors such as emotion regulation, social support, stigma perception, and personality traits may be related to help-seeking behaviors (25, 83, 84). Future research could adopt more comprehensive theoretical frameworks and longitudinal or experimental designs to further examine the complex associations among self-compassion, psychological factors, and psychological help-seeking delay.

## 5 Conclusion

This study integrates self-compassion theory into research on psychological help-seeking by examining help-seeking behaviors from an individual psychological perspective, thereby providing a novel perspective for understanding factors associated with psychological help-seeking delay. The findings indicate that self-compassion was significantly associated with psychological help-seeking delay, with mental health problems and negative academic emotions involved in the serial indirect associations between self-compassion and psychological help-seeking delay.

These findings provide new insights for educational institutions and families regarding potential psychological factors related to delayed help-seeking among college students. Future interventions may consider fostering self-compassion as one potential approach to promoting adaptive psychological functioning and encouraging timely engagement with professional psychological support. However, given the cross-sectional design of this study, causal relationships among self-compassion, mental health problems, negative academic emotions, and psychological help-seeking delay cannot be established. Future longitudinal research is needed to further examine the temporal relationships among these variables.

## Data Availability

The raw data supporting the conclusions of this article will be made available by the authors, without undue reservation.
